# Spatiotemporal Variations in Growth Rate and Virulence Plasmid Copy Number during *Yersinia pseudotuberculosis* Infection

**DOI:** 10.1128/IAI.00710-20

**Published:** 2021-03-17

**Authors:** Stephan Schneiders, Tifaine Hechard, Tomas Edgren, Kemal Avican, Maria Fällman, Anna Fahlgren, Helen Wang

**Affiliations:** a Department of Medical Biochemistry and Microbiology, Uppsala University, Uppsala, Sweden; b Department of Molecular Biology, Umeå University, Umeå, Sweden; Stanford University

**Keywords:** *Yersinia pseudotuberculosis*, type III secretion system, plasmid copy number, bacterial growth rate in tissues, ddPCR

## Abstract

Pathogenic *Yersinia* spp. depend on the activity of a potent virulence plasmid-encoded *ysc*/*yop* type 3 secretion system (T3SS) to colonize hosts and cause disease. It was recently shown that Yersinia pseudotuberculosis upregulates the virulence plasmid copy number (PCN) during infection and that the resulting elevated gene dose of plasmid-encoded T3SS genes is essential for virulence. When and how this novel regulatory mechanism is deployed and regulates the replication of the virulence plasmid during infection is unknown. In the present study, we applied droplet digital PCR (ddPCR) to investigate the dynamics of Y. pseudotuberculosis virulence PCN variations and growth rates in infected mouse organs. We demonstrated that both PCN and growth varied in different tissues and over time throughout the course of infection, indicating that the bacteria adapted to discrete microenvironments during infection. The PCN was highest in Peyer’s patches and cecum during the clonal invasive phase of the infection, while the highest growth rates were found in the draining mesenteric lymph nodes. In deeper, systemic organs, the PCN was lower and more modest growth rates were recorded. Our study indicates that increased gene dosage of the plasmid-encoded T3SS genes is most important early in the infection during invasion of the host. The described ddPCR approach will greatly simplify analyses of PCN, growth dynamics, and bacterial loads in infected tissues and will be readily applicable to other infection models.

## Abstract (precis)

Pathogenic *Yersinia* spp. depend on the activity of a potent virulence plasmid-encoded *ysc*/*yop* type 3 secretion system (T3SS) to colonize hosts and cause disease. It was recently shown that Yersinia pseudotuberculosis upregulates the virulence plasmid copy number (PCN) during infection and that the resulting elevated gene dose of plasmid-encoded T3SS genes is essential for virulence.

## INTRODUCTION

Bacterial pathogens have evolved various virulence strategies to successfully invade hosts and cause diseases. Pathogenic *Yersinia* deploys a potent plasmid-encoded *ysc*/*yop* type III secretion system (T3SS) that inhibits phagocytic cells and suppresses the inflammatory response (1, 2). *Yersinia* virulence requires an increased gene dosage of plasmid-encoded T3SS genes through elevated plasmid copy number (PCN). This recently discovered regulation of an independently replicating plasmid is an essential virulence mechanism in *Yersinia* (3). The actions of the T3SS and other virulence factors enable pathogenic *Yersinia* to evade the host immune response and proliferate in extracellular foci or microcolonies in lymphatic, liver, and spleen tissues (4–7).

While essential for virulence, deployment of the plasmid-encoded T3SS is metabolically expensive and pathogenic *Yersinia* species display calcium-dependent growth at 37°C due to the activity of the virulence plasmid-encoded T3SS (8). At 37°C in the absence of Ca^2+^, pathogenic *Yersinia* organisms switch on their T3SS and secrete massive amounts of bacterial toxins out of the cells (9, 10). Activation of T3SS secretion *in vitro* results in growth arrest and increased virulence PCN, and repression of T3SS (addition of excess Ca^2+^ at 37°C) restores bacterial growth.

Increased gene dose of virulence plasmid-borne genes was recently demonstrated to be essential for Yersinia pseudotuberculosis infection (3). A strain with a single copy of the virulence plasmid integrated into the chromosome (lacking PCN regulation) displayed decreased Yop expression, secretion, and translocation compared to wild-type bacteria and was avirulent in a mouse infection model. In contrast, an isogenic strain with three copies of the virulence plasmid integrated in the chromosome exhibited similar T3SS function and virulence compared to a wild-type strain. The single-copy variant showed temperature and Ca^2+^-independent growth *in vitro* while the isogenic three-copy strain displayed temperature and Ca^2+^-dependent growth *in vitro*. These results highlight the role of PCN variations in *Yersinia* T3SS function and demonstrate the high metabolic burden associated with increased gene dosage of virulence plasmid-borne genes during T3SS inductive conditions. Thus, the activity of the T3SS is paradoxical and pathogenic *Yersinia* must have evolved mechanisms to trade off essential virulence-associated metabolic costs to successfully spread and proliferate during infection.

Y. pseudotuberculosis is a gastrointestinal pathogen that usually causes self-limiting gastroenteritis in humans (11, 12), while high infection doses cause lethal systemic infection in mice (13, 14). Upon ingestion by mice, Y. pseudotuberculosis crosses the intestinal mucosal barrier, probably through microfold (M) cells in the intestinal epithelium, and proliferates in Peyer’s patches (PP) and the cecum. Some bacteria drain to the mesenteric lymph nodes (MLN), followed by systemic spread to the liver and spleen (15, 16). The virulence plasmid-encoded T3SS together with upregulation of PCN is essential for the efficient systemic spread giving rise to the lethal infection (3, 17), and T3SS-defective bacteria are still invasive but typically do not disseminate from the MLN (18).

**FIG 1:**
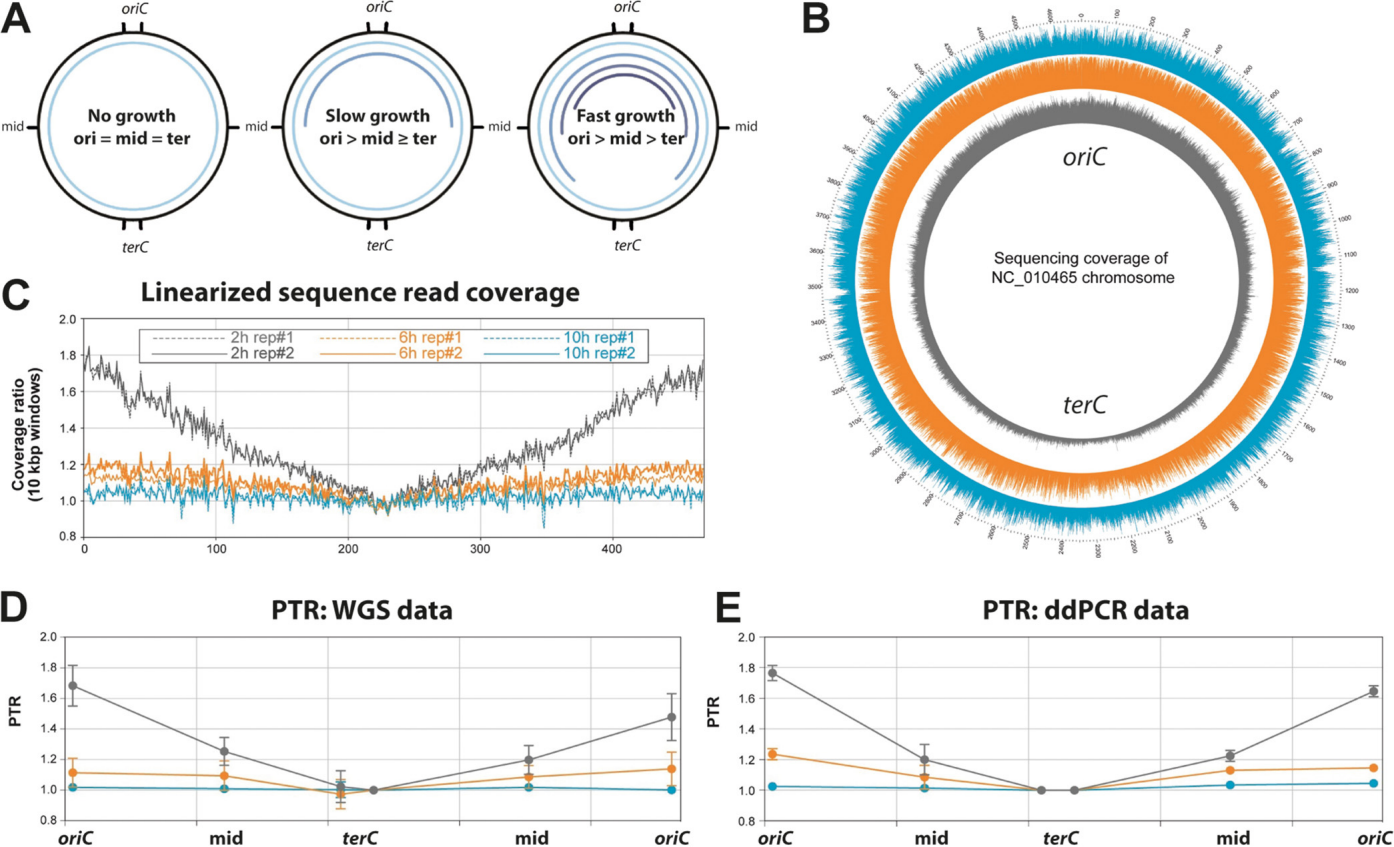
ddPCR enables precise determination of PTR ratio of bacterial populations. (A) Bidirectional replication of circular chromosome leads to an enhanced DNA dosage of regions that have already been passed by the replication fork. Bacterial populations show different patterns of DNA dosage in chromosomal regions depending on their growth rate. Outer black lines indicate the positions of primer pairs used in this study. Inner blue circle lines indicate the DNA dosage of chromosomal regions. (B) Sequence read coverage of Y. pseudotuberculosis DNA, isolated from a culture grown at 37°C with Ca^2+^, is plotted against a circular reference genome (*n* = 2). Gray, 2 h; orange, 6 h; blue, 10 h. (C) Coverage of the same samples plotted against linearized reference chromosome. (D) PTRs were calculated from the WGS data as the coverage depth at the position of the primers close to the origin divided by the coverage at the positions close to the terminus. (E) PTRs of the same samples determined by ddPCR. For panels D and E, the data represent the means ± the SD of two biological replicates.

Most bacteria bidirectionally replicate their circular chromosome from a single origin of replication (*oriC*). Therefore, within an actively replicating bacterial population, as the replication forks progress along the DNA molecule, the DNA dosage will be higher for genes closer to the *oriC* compared to genes closer to the replication terminus (*ter*) (Fig. 1A). For logarithmically growing bacteria, this difference in DNA dosage is evident in whole-genome sequencing (WGS) reads aligned to the circular chromosome, and a clear V-shaped coverage can be seen when the reads are aligned to the linearized chromosome (gray tracks in Fig. 1B and C). The growth rate of a bacterial population can be estimated by the ratio of the DNA coverage at the *oriC* divided by the coverage at the replication terminus (19, 20). This so-called peak-to-trough ratio (PTR) varies depending on the replicative status of the bacteria, and ratios of up to ∼2.7 have been reported for fast-growing Escherichia coli *in vitro* (19).

In the present study, we optimized a cost-efficient, sensitive, and accurate droplet digital PCR (ddPCR)-based method for determining PCN and PTR in Y. pseudotuberculosis. ddPCR is a third-generation qPCR method that allows absolute quantification of the target DNA molecules present in a sample (21–23). This method takes advantage of the random distribution of fragmented DNA in 20,000 water-oil emulsion droplets generated before the PCR (Bio-Rad). Each droplet is an independent PCR, and determining the number of positive droplets gives an absolute number of target DNA molecules present in the sample. The dynamic range of ddPCR is greatly improved compared to traditional qPCR methods (21), and the micropartitioning of the sample in the droplets effectively reduces the concentration of inhibitory factors. Thus, ddPCR allows more reliable and accurate detection of low abundant templates in heterogeneous samples compared to traditional qPCR methods without the need of a standard curve (22). The PCN variations and growth rates of heterogeneous *Yersinia* communities proliferating at different infection sites have not been investigated previously. Therefore, after validating our ddPCR methodology, we investigated how the PCN and growth rates vary in Y. pseudotuberculosis populations in different tissues in a murine infection model.

## RESULTS AND DISCUSSION

### Development and validation of ddPCR for determination of PCN and PTR in *Y. pseudotuberculosis*.

To validate our ddPCR method for establishing the PTR in Y. pseudotuberculosis, we determined the PTR of a Y. pseudotuberculosis YpIII/pIBX culture using both a PCR-free WGS approach (Fig. 1D) and ddPCR (Fig. 1E) with six different primer pairs targeting different regions of the chromosome (*oriC*, *mid*, and *ter* on either side of the *oriC*). The PTR of the Y. pseudotuberculosis culture ranged from a lowest value of ∼1 for bacteria in stationary phase, to a highest value of ∼1.8 at 2 h after inoculation. Critically, the PTR values calculated from ddPCR correlated with the PTR values determined by WGS of the same samples (Fig. 1D and E), validating the use of ddPCR for accurate PTR determination in bacterial cultures.

**FIG 2:**
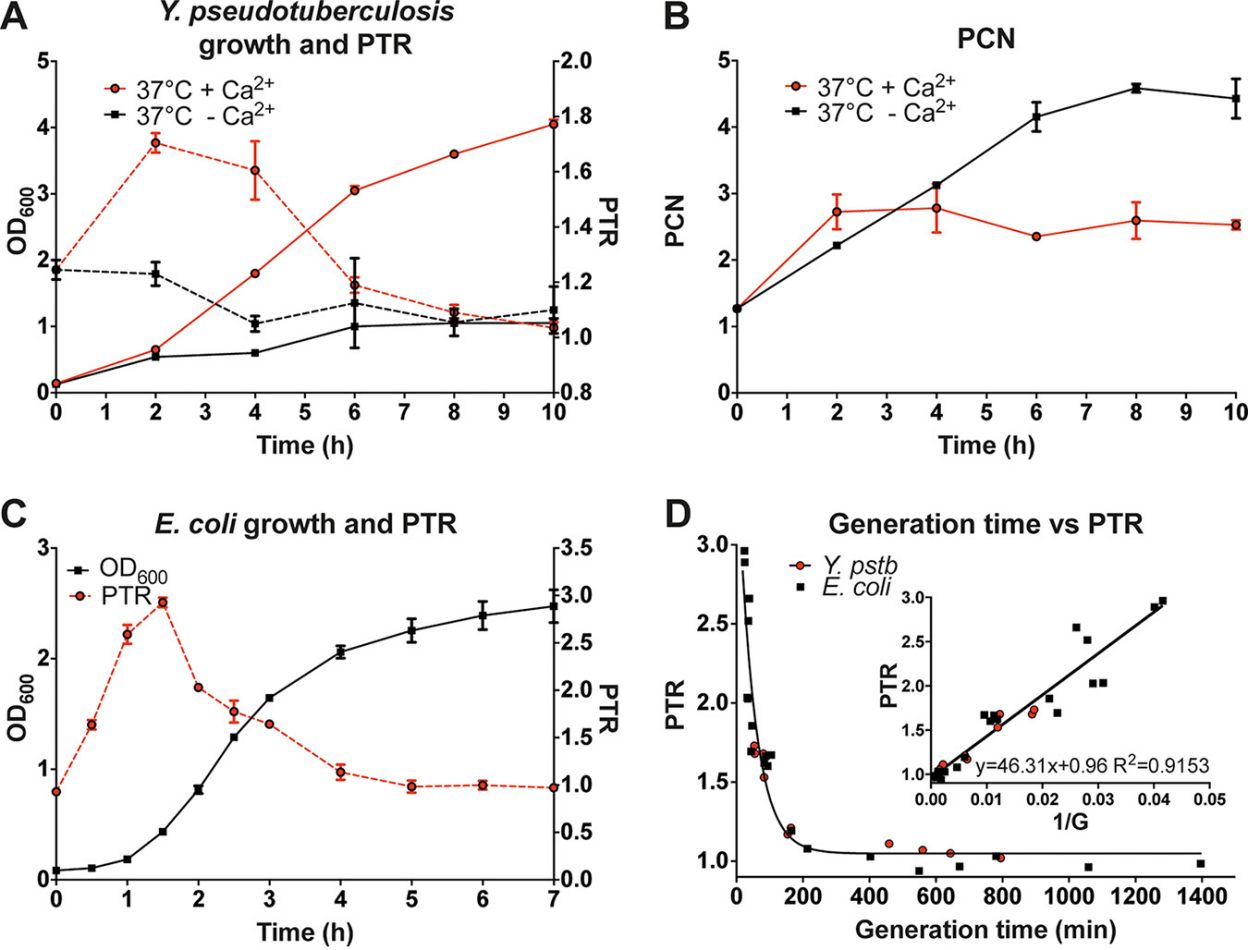
ddPCR-determined PTR ratios reflect *in vitro* growth rates of Y. pseudotuberculosis and E. coli. (A and B) T3SS-dependent upregulation of PCN results in a metabolic burden and inhibits Y. pseudotuberculosis growth. (A) PTRs (dashed lines) calculated based on ddPCR reflect the growth rate (solid lines) based on the OD of Y. pseudotuberculosis cultures grown under T3SS-repressive (37°C +Ca^2+^; red) and T3SS-induced (37°C −Ca^2+^; black) conditions. (B) PCN changes determined by ddPCR of the same cultures. (C) Growth and PTR measurements of E. coli grown in LB medium at 37°C. (D) PTR plotted against generation times in Y. pseudotuberculosis and E. coli. Data represent the means ± the SD of two biological replicates.

After verifying that the PTR of bacterial cultures could be determined by ddPCR, we next analyzed how the PTR related to PCN and growth in Y. pseudotuberculosis cultures grown at 37°C under T3SS-repressed (+Ca^2+^) and T3SS-induced conditions (–Ca^2+^). Pathogenic *Yersinia* species display calcium-dependent growth at 37°C due to the activity of the virulence plasmid-encoded T3SS (8). Activation of T3SS secretion *in vitro* results in growth arrest and increased PCN (black lines in Fig. 2A and B), and repression of T3SS (addition of excess Ca^2+^) restores bacterial growth and results in an increased PTR (see Fig. S1 in the supplemental material). The T3SS-repressed culture (+Ca^2+^) displayed a PTR of 1.6 to 1.8 *oriC* equivalents per cell compared to the replication terminus during logarithmic growth (Fig. 2A, red dashed line). On the other hand, the T3SS-induced culture (−Ca^2+^) showed limited growth, with a PTR value of 1 to 1.2 throughout the experiment (Fig. 2A, black dashed line). In the T3SS-repressed culture, the PCN initially increased and then settled after 2 h to ∼2.5 virulence plasmid equivalents per cell, while in the T3SS-induced culture, the PCN increased to ∼5 (Fig. 2B). We observed an inverse relationship between PTR and PCN *in vitro*; when the T3SS is active, *Yersinia* turns on PCN, and growth slows down.

### PTR values reflect the growth rates of different *Enterobacteriaceae* with different generation times.

To further investigate the relationship between PTR and growth rates in different *enterobacteriaceae*, we determined the PTR and generation times of Y. pseudotuberculosis and E. coli cultures. When grown in Luria-Bertani (LB) medium at 37°C, Y. pseudotuberculosis and E. coli have different growth rates with doubling times of around 60 and 20 min, respectively. We found that the calculated fastest generation times were 54 and 24.5 min (Fig. 2A and C) during early log phase for Y. pseudotuberculosis and E. coli (MG1655 [24]) cultures, respectively. Independently of the growth rate, the plot of PTR values against calculated generation times fit an exponential one-phase decay curve (Fig. 2D). Inverting the generation time, yielded a linear model (Fig. 2D, inset). Thus, the relationship between PTR and growth rate was similar in both species, indicating that PTR can be used as a proxy for determining the growth rate of entero-bacterial populations.

### ddPCR is a robust method for determination of PCN and PTR in complex samples with interfering DNA.

**FIG 3:**
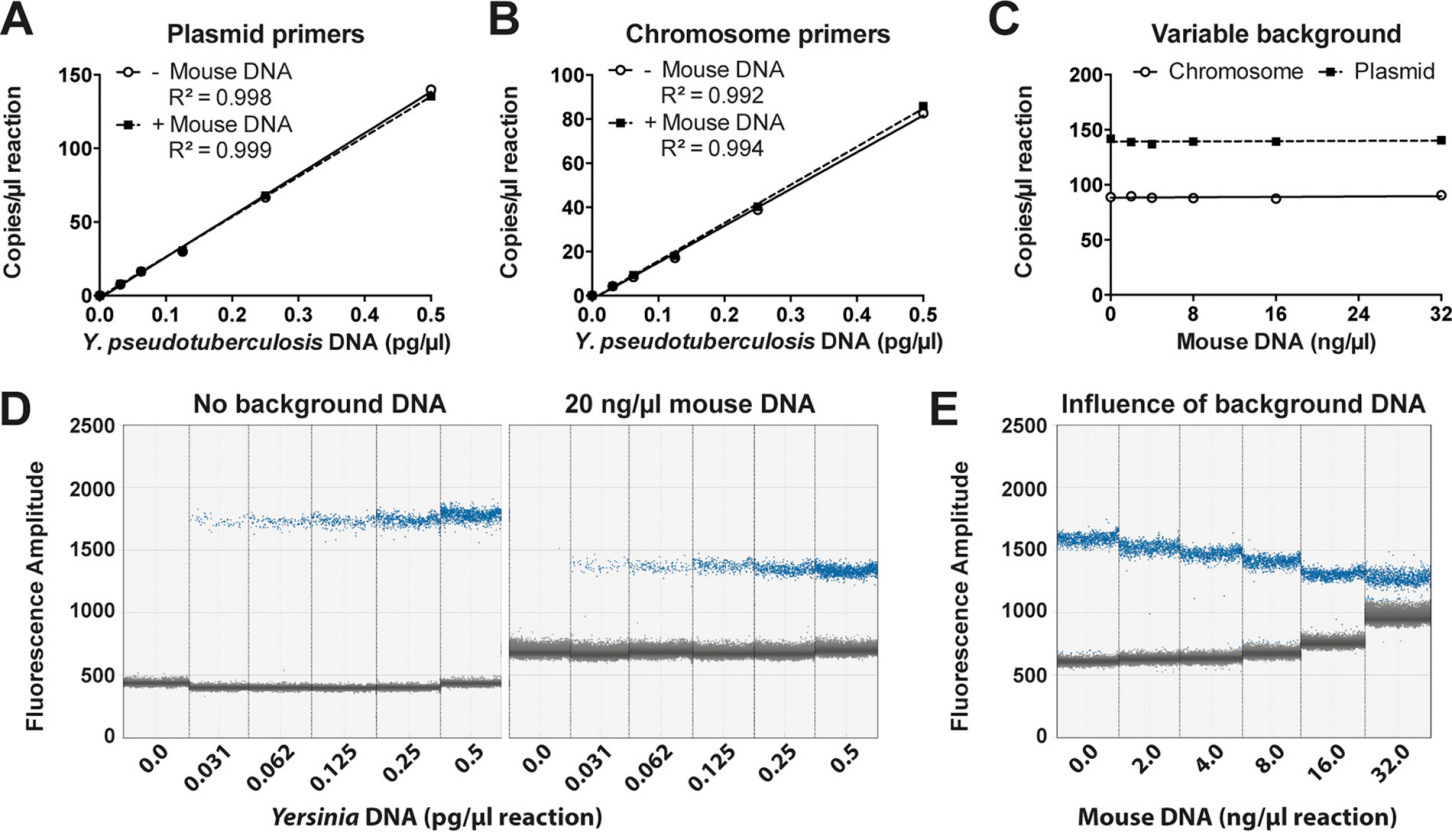
ddPCR accurately determines the PTR ratio and PCN within a dynamic range of target and background DNA. A ddPCR method using Y. pseudotuberculosis DNA isolated from a culture grown to stationary phase at 26°C was validated. (A and B) Plasmid and chromosome quantities in a dilution series of Y. pseudotuberculosis DNA, with or without 20 ng/μl mouse DNA as background. (C) Plasmid and chromosome quantities from 0.5 pg/μl Y. pseudotuberculosis DNA with increasing levels of mouse DNA as background. (D and E) Separation pattern of positive (blue) and negative (gray) droplets with increasing concentrations of Y. pseudotuberculosis DNA, with or without 20 ng/μl mouse DNA as background (D), and of 0.5 pg/μl Y. pseudotuberculosis DNA, with increasing concentrations of mouse DNA as background (E).

These initial experiments were performed using DNA isolated from laboratory grown bacterial cultures that lacked interfering host DNA. Using traditional WGS techniques, it is challenging to determine accurate PTR and PCN values for invasive bacteria in infected tissues due to the small amounts of bacterial DNA compared to host DNA present in the sample. Each diploid eukaryotic cell contains ∼1,000 times more genomic content compared to a bacterial cell—effectively diluting out the bacterial reads in WGS data. To further validate our ddPCR method, we tested the dynamic range of the assay in both the presence and absence of murine DNA (Fig. 3; see also Fig. S2A and B). The assay was robust with respect to foreign DNA, and could accurately detect the target bacterial DNA in the presence of an at least 800,000-fold excess of murine DNA (32 ng/μl mouse DNA in Fig. 3E), using both the virulence plasmid primer pairs and chromosomal primer pairs. Externally added murine DNA in the sample did not affect the dose dependency of the assay or reduce the absolute detection of target DNA (Fig. 3). However, the dynamic range of traditional qPCR using the same primer pairs was much lower than ddPCR (see Fig. S3). The reliable detection limit of target DNA was 0.25 pg/μl using qPCR (see Fig. S3A), and lower concentrations deviated significantly from the linear range of the standard curve. This was most likely due to nonspecific products appearing after quantitation cycles (*C_q_*) 35 (see Fig. S3B). Importantly, high concentrations of foreign DNA had a huge impact on the *C_q_* of target DNA (see Fig. S3C). When using small amounts of target DNA (0.5 pg/μl of *Yersinia* DNA), no reliable detection can be achieved with concentrations above 1.5 ng/μl mouse DNA present in the sample (see Fig. S3C, inset). With our ddPCR method, increasing total double-stranded DNA (dsDNA) present in the sample was associated with increased background fluorescence, making it more difficult to differentiate between negative and positive droplets when using high total DNA concentrations (see Fig. S2C). We were able to differentiate between positive and negative droplets with up to 32 ng/μl mouse DNA in the sample, corresponding to 640 ng of total mouse DNA in the sample (Fig. 3E). These results showed that ddPCR is a sensitive and robust method, with a high dynamic range enabling precise determination of target DNA, even in complex samples with a high excess of interfering foreign DNA.

### Determination of PCN and PTR in different tissues in a murine infection model.

**FIG 4:**
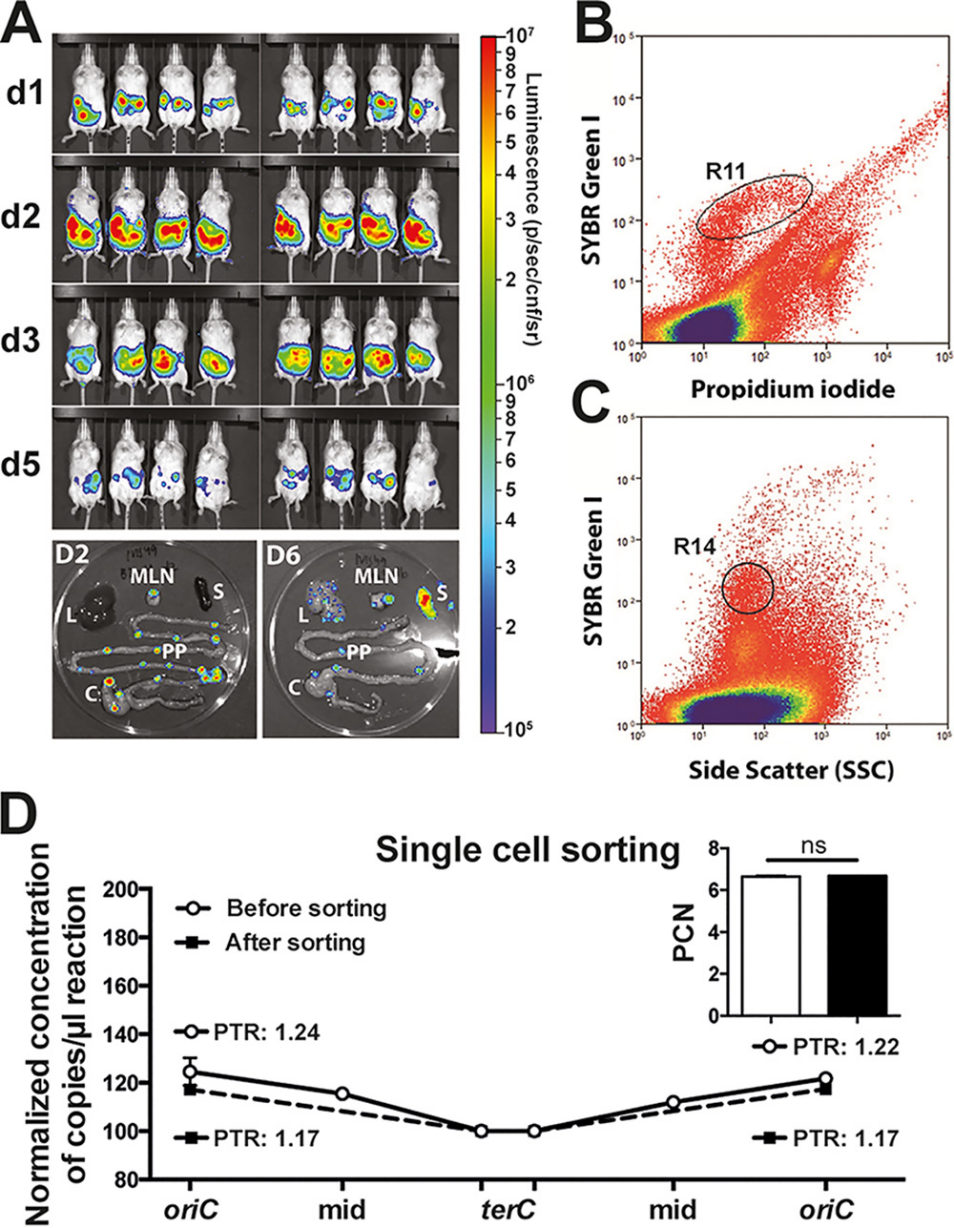
Mouse infections and single cell sorting. (A) IVIS images of mice infected with Y. pseudotuberculosis. Colors indicate the levels of light emitted by bioluminescent bacteria. BALB/c mice (Charles River, 8-week-old, females) were orally infected with an infection dose of 2 × 10^8^ CFU/ml. At the indicated days after infection, mice were sacrificed, and infected organs were dissected for DNA extraction and ddPCR analysis. Isolated organs: PP, Peyer’s patches; MLM, mesenteric lymph nodes; C, cecum; L, liver; S, spleen. (B and C) Scatterplots of events collected during sorting of homogenized and filtered PP cells taken 2 days postinfection, stained with SYBR green I and PI. (B) Gating of SYBR green I (+) and PI (–) cells to indicate live cells (R11). (C) A narrow “cells” gate with moderate SYBR green I staining (R14) to exclude unwanted events of nonspecific SYBR green staining. (D) Plotted coverage of normalized DNA quantity of the PP sample before sorting (white symbol; solid line) and after sorting (black symbol; dashed line) using ddPCR. (Inset) PCN of the same sample before and after sorting. Data represent the means ± the SD of two technical replicates (ns, not significant [Student *t* test]).

To investigate the spatiotemporal variation of PCN and growth rates during infection, we infected mice with a virulent Y. pseudotuberculosis strain using an oral mouse infection model (3). Mice were infected with 2 × 10^8^ CFU (CFU/ml of the bioluminescent Y. pseudotuberculosis YpIII/pIBX strain) (16) via their drinking water. Y. pseudotuberculosis YpIII/pIBX contains a mini-Tn*5 luxCDABE* inserted in the native pCD1 virulence plasmid, enabling visualization of bioluminescent bacteria in the infected organs using an *in vivo* imaging system (IVIS) (Fig. 4A). At different postinfection time points, bioluminescent PP, ceca, MLN, spleens, and livers were collected from infected mice. The larger organs (ceca, spleens, and livers) were further dissected to isolate the bioluminescent bacterial foci in an effort to increase the amount of bacterial DNA compared to host DNA present in the samples.

We first conducted a control experiment designed to evaluate the accuracy of our ddPCR methodology in infected tissues. From infected PPs, bacterial cells were sorted from host cells using a MoFlo EQ flow cytometer (Beckman Coulter). The homogenized and filtered sample was stained with SYBR green I and propidium iodide (PI), enabling differentiation of PI-impermeable bacterial cells from dead eukaryotic cells and cell debris in the sample (Fig. 4B and C). To narrow down the sort region of live cells, a combination of gates R11 and R14 was used. The R11 gate in the bivariate SYBR green versus the PI staining plot (Fig. 4B) includes cells that are positive for SYBR green and negative for PI staining (dead cells). The R14 gate (Fig. 4C) was used as a more stringent live “cells” gate for SYBR green staining where cells had appeared taking different scatterplots in consideration (all not shown). Hence, this could limit events that ended up in the R11 gate only based on SYBR green fluorescence such as nonspecific staining of cell and noncell debris.

A total of 24,141 bacterial cells were sorted, and we verified successful gating for *Yersinia* cells using microscopy and ddPCR quantification of estimated cell events. Importantly, we obtained similar PCN and PTR values from DNA isolated from the sorted cell population compared to DNA purified directly from the homogenates before sorting (Fig. 4D). This indicated that our ddPCR method could be used to accurately determine both the PCN and PTR in total DNA isolated from infected organs.

**FIG 5:**
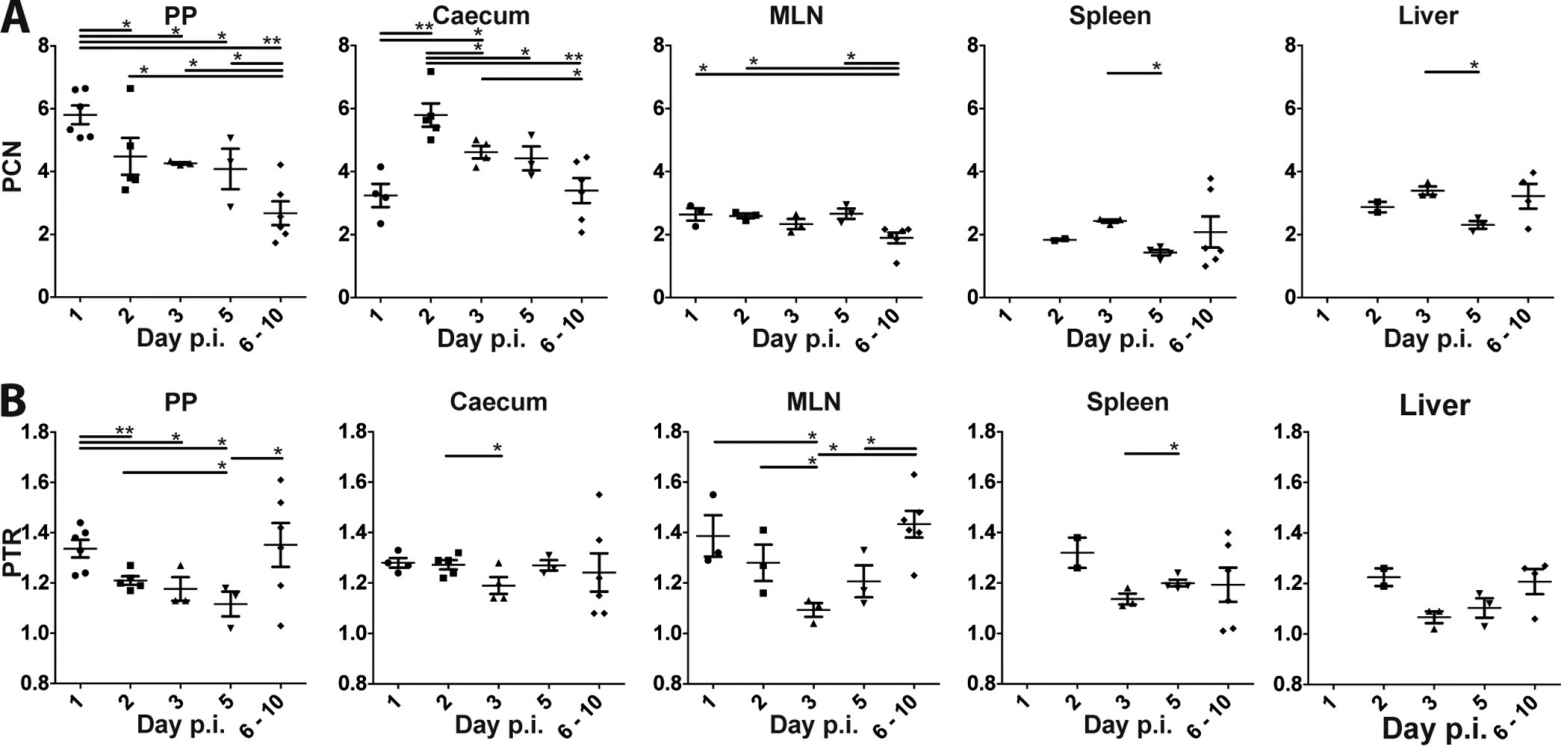
Spatiotemporal variations of PTR and PCN in mice during Y. pseudotuberculosis infection. (A) Virulence PCN over a 10-day time period. PCN was calculated as the ratio between concentration of plasmid and chromosome DNA using ddPCR. (B) PTR in different organs and at different time points postinfection (p.i.). PTR was calculated as the ratio between DNA dosage of chromosomal regions located at *oriC* and *ter* using ddPCR. The data represent the means ± the SEM of at least two biological replicates (*, *P* ≤ 0.05; **, *P* ≤ 0.01 [Mann-Whitney U test]).

In PP at 1 day after infection, the PCN was ∼6. This PCN level decreased with progressive infection, reaching ∼2.5 in terminally ill mice that were euthanized at 6 to 10 days after infection (Fig. 5A). The initial high PCN levels were similar to those previously reported using TruSeq WGS of PP at 2 days after infection (3). In ceca, the PCN showed a similar but delayed trend, with a value of ∼3 at 1 day postinfection, and a peak at ∼6 at 2 days postinfection. Again, this PCN level decreased, and was ∼3 in mice euthanized at 6 to 10 days postinfection. MLN samples showed a different trend, with the PCN remaining ∼2.5 throughout infection, similar to the level observed in T3SS-repressed bacteria grown at 37°C. This is in line with previous studies showing that Y. pseudotuberculosis with a defective or absent *ysc*/*yop* T3SS can proliferate in MLN (18). The deeper systemic organs also exhibited a relatively low PCN throughout the infection, with values of ∼2 in spleens and ∼3 in livers. These findings are interesting since a functional T3SS is required for systemic spread of Y. pseudotuberculosis in mice (17). Moreover, these systemic sites seem to be colonized later in infection, and no bacteria were detected at 1 day postinfection and only two positive samples were identified at 2 days postinfection. It is possible that the bacteria have established heterogeneous subpopulations such as microcolonies by the time bioluminescent foci are detected in these organs and the T3SS is required early in the colonization/infiltration of these organs. In fact, how bacteria invade these systemic organs is not clear and both direct dissemination from the GI tract and spread from MLN have been proposed (14). Overall, we found that the PCN values at early infection sites (PP and cecum) were initially high, and these levels decreased over time with progressive infection, i.e., after the bacteria had crossed the mucosa-associated lymphoid tissue and invaded deeper tissues (MLN, spleen, and liver). This suggests that high virulence PCN is probably most important during the clonal colonization phase compared to in later infection stages. Importantly, our results show that the PCN is not static during infection. The different PCN values in different tissues and over time indicate that the virulence PCN of *Yersinia* is regulated in response to environmental stimuli in different tissues.

We determined the PTR values of the infected samples using primer pairs proximal to the origin of replication and the replication terminus. In all examined samples, the PTR was between 1.1 and 1.4 (Fig. 5B), corresponding to generation times of ∼600 and ∼162 min, respectively. In comparison, we found PTR values of ∼1.1 and ∼1.8 for *Yersinia* grown *in vitro* under T3SS-induced and T3SS-repressed conditions, respectively (Fig. 2A). The recorded relatively low replicative rates during infection were expected, mirroring the struggle of invading bacteria to proliferate in the hostile host environment. We observed a tendency toward increased PTR values in organs from mice showing severe signs of infection and euthanized on days 6 to 10, compared to at 5 days postinfection. This indicates that the bacteria could proliferate faster in terminally ill mice after overcoming the initial host immune onslaught. We also observed a tendency for higher PTR values (∼1.4) in PP and MLN at 1 day postinfection, which decreased to the minimum value at 5 days postinfection in PP, and 3 days postinfection in MLN. Overall, the growth rates as determined by PTR varies among different organs and over time, indicating that the bacterial populations confronted different microenvironments during the course of infection.

### *Y. pseudotuberculosis* displayed spatiotemporal variations in growth rate and virulence PCN and adapt to different microenvironments during infection.

During infection, we saw no clear relationship between PCN and PTR compared to the inverse relationship seen in laboratory grown *Yersinia* cultures. Bacteria proliferating in PP at 1 day postinfection displayed high PCN values (∼6), as well as high replicative rates (PTR of ∼1.4). A similar decoupling of PCN and replication was also observed in the cecum at 2 days postinfection, where the PTR value was ∼1.3 and the PCN values were also high. In the deeper tissues, the low PCN values recorded in MLN were associated with high replicative rates early in the infection (PTR of ∼1.4 at 1 day postinfection), followed by slower growth at 3 days after infection. These data indicated that the T3SS-dependent growth restriction was not as evident during infection compared to in bacteria grown *in vitro*. This suggests that the invading pathogen could have evolved mechanisms to trade-off virulence-associated fitness costs to support proliferation in the hostile host environment.

In addition, the quantitative nature of ddPCR enables determination of the bacterial loads in the infected organs (see Fig. S4). For our analyses, we isolated total DNA from whole PP and MLN, as well as DNA from dissected parts with bioluminescent foci from the larger organs (cecum, liver, and spleen). Early in the infection, we observed a delayed correlation between bacterial growth rates (PTR) and relative bacterial loads. For the first 3 days after infection, high PTR values were recorded, and the consecutive time points showed increased bacterial loads. Later in the infection (day 5 and days 6 to 10), there was no clear relationship between PTR and bacterial loads. This discrepancy can most likely be explained by our analysis of only a portion of the dissected systemic organs in which bacterial growth would be expected at later time points. The bacteria could also have diversified and different subpopulations proliferating in various niches inside the organs are too heterogenous to discern any clear relationship in later time points. The recorded bioluminescence emitted from whole mice in the IVIS images showed a similar trend, with a peak at 2 days after infection, followed by a decrease over time (Fig. 4A).

The observed spatiotemporal variations in PCN and PTR among different organs and over time show that *Yersinia* colonization and infection is a dynamic process, during which bacteria adapt to different environments. Enteric *Yersinia* species exhibit lymphoid tropism—colonizing PP, cecum, and MLN tissues (25). Within these tissues, extracellular microcolonies are established in which the bacteria facing the host environment are exposed to phagocytes, while other bacteria are buried inside the microcolony. These different bacterial populations are clearly heterogeneous and display distinct gene expression profiles, including expression of T3SS effectors (5, 25).

The ddPCR results presented in this study represent a snapshot of the average growth rate and PCN of all bacteria present in the sample at the time of dissection. Thus, the method is unable to resolve variations at the single cell level or heterogeneities in bacteria occupying different niches in the infected organ. The high variability in growth rates accompanied with stable PCN over time in analyzed MLNs indicate that bacteria proliferating in this organ adapt and respond to changes in their environment independent of changes in T3SS. A previous report shows that *Yersinia* cured of the virulence plasmid and thus lacking the T3SS resides and proliferates extracellularly in B and T cell-rich regions of the MLN (18). The low PCN levels found in the MLN support these findings and indicate that high T3SS activity is not necessary for bacteria to colonize this organ.

Our present findings support a model in which invading clonally expanding bacteria display high PCN and relatively high replicative rates early in the infection. Importantly, our data demonstrate that *Yersinia* replicates throughout the infection in all examined organs. As the bacteria proliferate and establish microcolonies, the relative importance of the T3SS decreases, which is reflected by the decreased PCN over time. The observed slower growth is most likely due to a combination of bacterial containment by the host immune system and nutritional limitation in the colony. The trend toward increased growth rates in terminally ill euthanized mice likely reflects the spread and proliferation of free bacteria as the infection progress. The dynamic changes of PCN in different infection sites has most likely evolved as a regulatory tactic to trade off virulence costs during infection to maximize proliferation of *Yersinia* in the host. Regulation of plasmid-encoded functions by gene dosage variations could be a general mechanism used by other bacteria in response to environmental cues.

## MATERIALS AND METHODS

### Bacterial strains and growth conditions.

Y. pseudotuberculosis YpIII/pIBX (16) and E. coli MG1655 (24) were routinely grown in LB broth or agar at 26°C for Y. pseudotuberculosis and 37°C for E. coli. Routine Y. pseudotuberculosis cultures were supplemented with 50 μg/ml kanamycin for maintenance of the pIBX virulence plasmid. For T3SS induction experiments, overnight Y. pseudotuberculosis cultures were inoculated in fresh LB medium (1:20) supplemented with 5 mM CaCl_2_ (T3SS repressed conditions, +Ca^2+^) or 20 mM MgCl_2_ and 5 mM EGTA (T3SS induced conditions, –Ca^2+^), followed by 1 h incubation at 26°C before shifting the cultures to 37°C. All growth analyses were carried out in duplicates and the bacterial densities were determined by measuring the optical density at 600 nm (OD_600_).

### DNA purification.

Tissue samples ranging in weight between 44 and 138 mg were homogenized 2 × 55 s in 1-ml sterile phosphate-buffered saline (PBS) using a gentleMACS Octo Dissociator (Miltenyi Biotec). Total DNA from bacterial cells and infected tissues was purified using a GeneJet Genomic DNA purification kit (Thermo Scientific) according to the manufacturer’s recommendations for Gram-negative bacteria and mammalian tissues, respectively. The final concentration of dsDNA was determined using a Qubit 3.0 fluorometer according to manufacturer’s recommendations (Thermo Scientific).

### ddPCR.

ddPCR analyses were carried out with EVA-Green using the Bio-Rad ddPCR QX200 system according to manufacturer’s recommendations. Briefly, 12.5 μl of 2× EVA-Green master mix (Bio-Rad), 1 μl of HindIII-HF (NEB), 10.5 μl of template DNA, and 1 μl of respective primer pairs (200 nM final concentration; see Table S1 in the supplemental material) were mixed and distributed into a 96-well qPCR plate (Bio-Rad). The plate was incubated for 10 min at room temperature to digest the target DNA before droplets were generated in an Automated Droplet Generator (Bio-Rad). PCR was performed with a hot-start/enzyme activation at 95°C for 5 min, denaturation at 94°C for 30 s, and amplification at 58°C for 1 min over 40 cycles, followed by signal stabilization at 4°C for 10 min and 90°C for 5 min. For all steps, a ramp rate of 2°C/s was used. Subsequently, the droplets were analyzed in the QX200 droplet reader (Bio-Rad). The data were analyzed with QuantaSoft Analysis Pro 1.0.569 (Bio-Rad).

### Quantitative PCR.

Quantitative PCRs were performed using a Bio-Rad CFX96 real-time system according to manufacturer’s recommendations. Briefly, a 20-μl solution containing 10 μl of Master Mix PerfeCTa SYBR green FastMix (Quantabio), 1 μl of respective primer pairs (200 nM final concentration), and 9 μl of template DNA with or without variable amounts of mice DNA were mixed and distributed into a 96-well quantitative PCR plate. Reactions were conducted in triplicate. PCR cycling was performed using a two-step cycling protocol with the following parameters: hot-start at 95°C for 5 min, followed by 40 cycles of 95°C for 30 s and 55°C for 30 s, followed by a standard melting-curve analysis from 65 to 95°C at 0.5°C increments per cycle with a ramp rate of 0.5°C/s. The data were visualized and processed using CFX Manager 3.1 (Bio-Rad).

### Whole-genome sequencing and data analysis.

Purified total DNA was sequenced using the Illumina TruSeq DNA PCR-free protocol by Novogene. The sequencing reads were aligned to Y. pseudotuberculosis reference sequences (NC_010465 for the YPIII chromosome and an in-house pIB1 sequence for the plasmid) using the CLC Genomic Workbench 11 (CLC Bio; Qiagen). The coverage for single nucleotides was calculated and the average coverage in a 10-kb window was plotted for visualizations. The PCN and PTR were calculated by dividing the average coverage of plasmid DNA with the average coverage of chromosomal DNA and the average coverage at *oriC* with the average coverage at *ter* of the mapped reads, respectively. The sequence data used in this study have been deposited in the European Nucleotide Archive (ENA) under accession number PRJEB38239.

### Mouse infection and bioluminescent imaging.

Eight-week-old female BALB/c mice (Charles River) were allowed to acclimate to the new environment for 1 week before the experiments. Mice were deprived of food and water for 16 h prior to oral infection with bioluminescent wild-type YpIII/pIBX. Bacteria grown overnight at 26°C were harvested and resuspended at a concentration of 2 × 10^8^ CFU/ml in sterile tap water supplemented with 150 mM NaCl. The bacterial suspensions were provided for 6 h as drinking water. The infection dose was determined by viable counts and drinking volume. Mice were inspected frequently for signs of infection, and mice showing prominent clinical signs were euthanized promptly to prevent suffering. Mice were monitored for bioluminescent emission using IVIS Spectrum (Caliper LifeSciences). Mice were anesthetized using the XGI-8 gas anesthesia system (Caliper LifeSciences) prior to imaging with 2.5% IsoFluVet in oxygen (Orion Pharma Abbott Laboratories, Ltd., Great Britain) and during imaging in 0.5% IsoFluVet. Images were acquired and analyzed using Living Image 4.5 (Caliper LifeSciences). To analyze bacterial localization within organs, mice were euthanized. Then, the intestines, mesenteric lymph nodes, livers, and spleens were removed, and the organs were imaged by bioluminescent imaging. Bioluminescent regions of the larger organs (cecum, liver, and spleens) were further dissected before freezing in liquid nitrogen.

Mice were housed in accordance with the Swedish National Board for Laboratory Animals guidelines. All animal experiments were approved by the Animal Ethics Committee of Umeå University (Dnr A65-15).

### FACS analysis and single cell sorting.

A Peyer’s patch sample from 2 days after infection, was homogenized in 1 ml of PBS for 2 × 55 s on a gentleMACS Octo Dissociator (Miltenyi Biotec) using a C-Tube (Miltenyi Biotec), which allows dissociation of viable single cells from tissue samples. Single cells were isolated by filtering through a 30-μm nylon mesh. Here, an aliquot of the filtered sample was diluted 200 times, and live/dead staining was performed. For this purpose, samples were incubated for 10 min with SYBR green I and PI to final concentrations of 1 and 2 μM, respectively.

The sorting was performed with a MoFlo Astrios EQ flow cytometer (Beckman Coulter) using the 488-nm laser and 530/40-nm filter (SYBR green I; all cells), 532-nm laser and 622/22-nm filter (PI; dead cells) for excitation and emission, plus a 70-μm nozzle, a sheath pressure of 60 lb/in^2^, and 0.1-μm-filtered 1× PBS as sheath fluid. Forward scatter was used as the trigger channel. A tube sorting of single cells was performed with the most stringent settings (purify mode and 1- to 2-drop envelope) in order to obtain both a qualitative and quantitative measurement of the sorted sample. Cell sorting was performed at the Microbial Single Cell Genomics Facility at Science for Life Laboratory in Uppsala.

### Generation time calculations.

The generation times of bacterial cultures were calculated from the 37°C growth curves presented in Fig. 2A and B for the T3SS repressed Y. pseudotuberculosis culture grown in the presence of Ca^2+^ (solid red line in Fig. 2A) and E. coli (solid black line in Fig. 2C) using the following formula:

$$G_{\text{min}}=\frac{t_{\text{min}}}{3.3\times {\text{logOD}}_{t2}/{\text{OD}}_{t1}},$$

where *t*_min_ is the time interval in minutes, OD*_t_*_1_ is the OD_600_ at the beginning of the time interval, and OD*_t_*_2_ is the OD_600_ at the end of the time interval. Each calculated value from the biological duplicates was plotted against the corresponding PTR value at OD*_t_*_2_. The resulting data fit a single exponential one-phase decay curve with a *R*^2^ value of 0.8872. A linear model was constructed by inverting the generation times (1/*G*), and both data sets fit a single line with the equation *y* = 46.31*x* + 0.96, with an *R*^2^ value of 0.9153.

### Quantification and statistical analysis.

Statistical analysis details of each individual experiment are presented in the figure legends or relevant methods section. Statistical analyses were performed using GraphPad Prism 6.

### Data availability.

Sequence data used in this study have been deposited in the European Nucleotide Archive under accession number PRJEB38239.

## Supplementary Material

Supplemental file 1
