# Supplementary material for: Spatiotemporal Variations in Growth Rate and Virulence Plasmid Copy Number during Yersinia pseudotuberculosis Infection
Source: Infect Immun. 2021 Mar 17;89(4):e00710-20. doi: 10.1128/IAI.00710-20 (PMC8090943; doi:10.1128/IAI.00710-20)
Supplement: Supplemental file 1 [file IAI.00710-20-s0001.pdf]

Supplemental material

Fig. S1

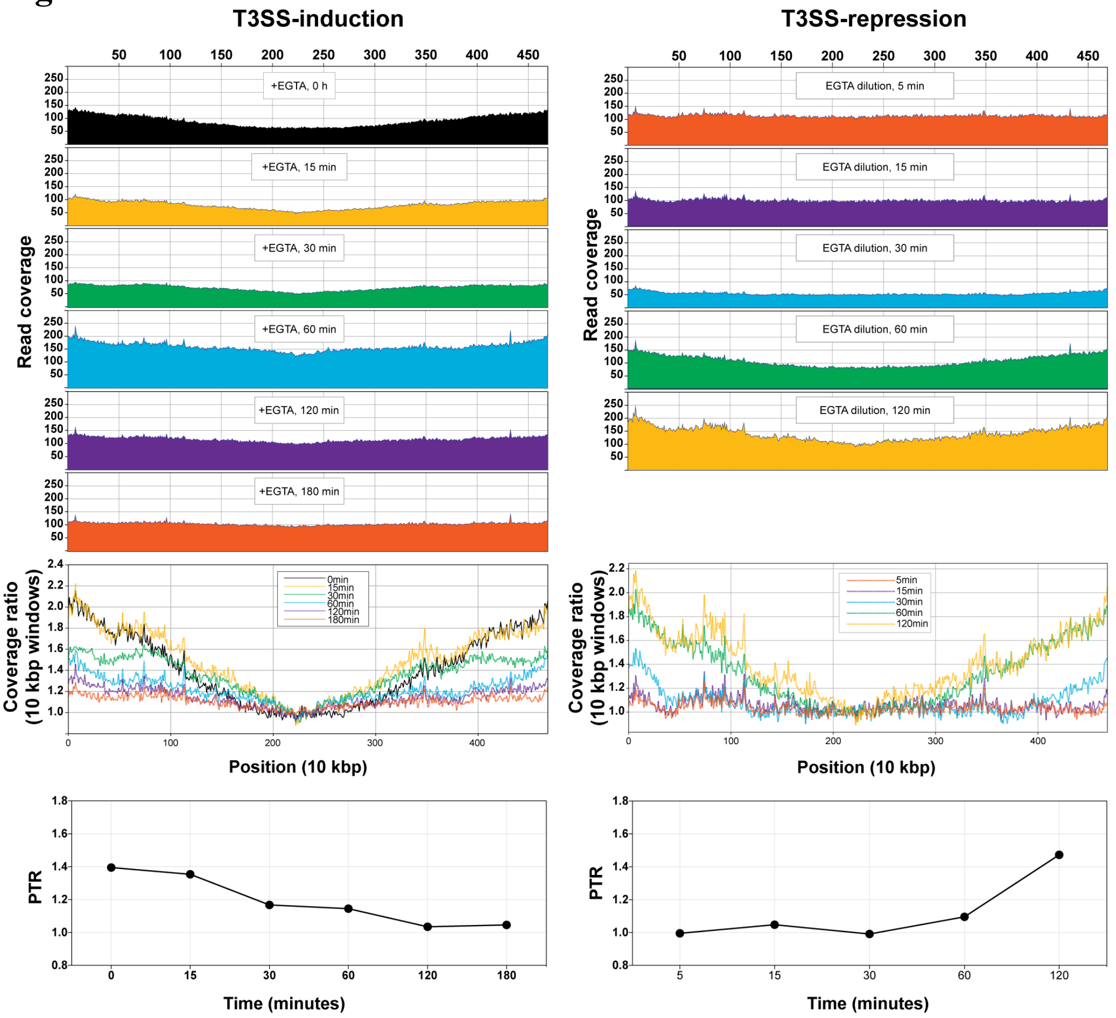

Fig. S1 | PTR of *Y. pseudotuberculosis* culture decreases under T3SS induction and can be reversed followed by repression of T3SS. Log-phase *Y. pseudotuberculosis* was shifted to T3SS-induced condition (37°C −Ca^{2+}, "+EGTA") for 3 hours, followed by addition of excess of Ca^{2+} (restoration of T3SS-suppression, "EGTA dilution"). Samples were taken at indicated time intervals and DNA was extracted and whole genome sequenced. Coverage depth was plotted against linearized reference chromosome. PTRs were calculated based on ddPCR methods describe above.

Fig. S2

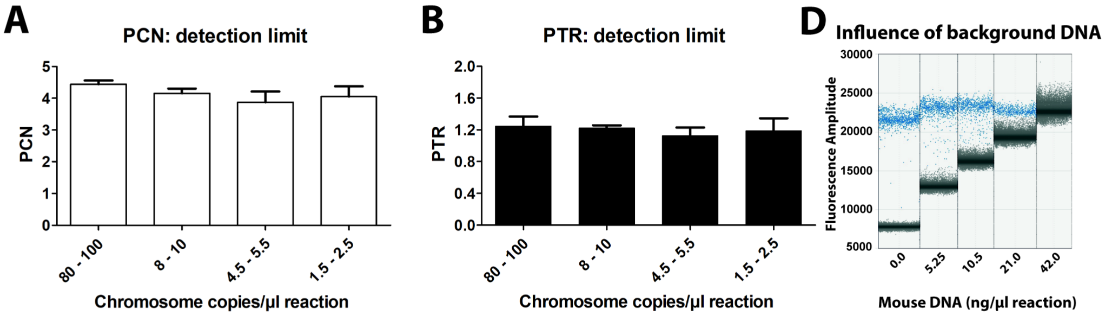

Fig. S2 | Determination of ddPCR detection limits. A.B, To determine the lower limit of target copies, dilution series of a Peyer's patch sample taken 3 days post-infection (p.i.) were used to calculate the PTR and PCN. Data represent the mean ± SEM of two technical replicates. No significant differences were found by Student's t-test. **C,** Separation pattern of 0.42 pg/μl *Y. pseudotuberculosis* DNA with an increasing concentration of mouse DNA as background.

Fig. S3

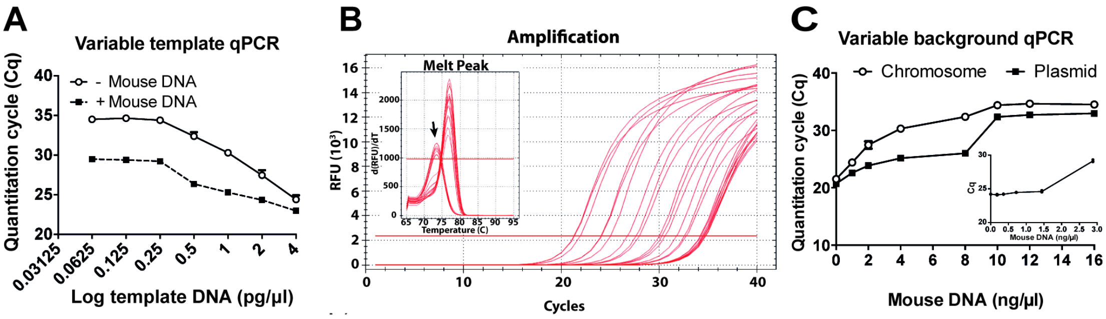

Fig. S3| Assessment of the dynamic range of qPCR in samples with low target concentrations and high background DNA. A.B, Validation of qPCR method using *Y. pseudotuberculosis* DNA isolated from a culture grown to stationary phase at 26°C. **A**, qPCR standard curves generated with two-fold dilutions of *Y. pseudotuberculosis* DNA using a chromosomal primer pair, with and without 20 ng/μl mouse DNA in the reaction. **B**, Amplification traces and melt-curves (insert) of qPCR reactions with two-fold dilution series of *Y. pseudotuberculosis* DNA using a chromosomal primer pair without background mouse

DNA. Arrow shows appearance of unspecific amplification at Cq values above 35. **C**, Validation of Cq values derived from a qPCR experiment with 0.5 pg/µl *Y. pseudotuberculosis* DNA in the presence of increasing concentrations of mouse DNA. Insert shows that reliable detection of 0.5 pg/ul *Y. pseudotuberculosis* DNA can be achieved using the chromosomal primer pair with concentrations up to 1.5 ng/ul mouse DNA present in the sample. Data represents the mean ± SD of technical triplicates.

Fig. S4

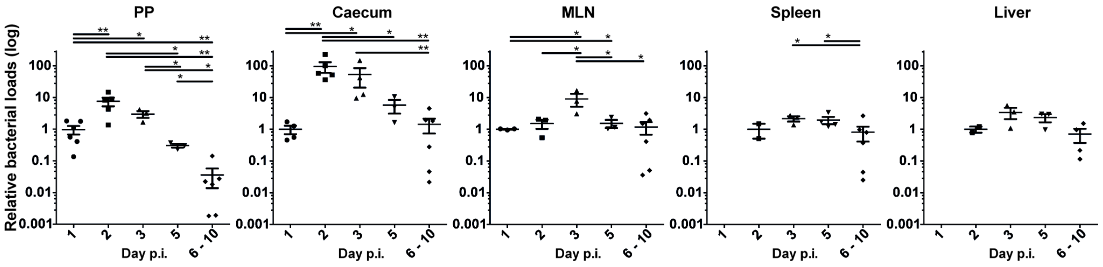

Fig. S4 | Determination of bacterial loads in infected mouse organs via ddPCR. The bacterial load per mg of infected organ tissue was calculated based on the copy number of chromosomal regions close to the terminus. Data were normalized against the value from first day post infection to show the relative changes of bacterial load during infection. Data represent the mean of at least two biological replicates (*$P \leq 0.05$; **$P \leq 0.01$, Mann-Whitney U test).

Table S1. List of primers used in this study. (C = Chromosome, P = Plasmid, O = Origin, M = Middle, T = Terminus, L = Left, R = Right)

Primer sets for *Y. pseudotuberculosis*

Name	Direction	Sequence 5' - 3'
C, O, L	Fw	GACCCAATCGCGACGTTTAC
	Rv	CCATCTTGCCAACGGGTAGTG
C, M, L	Fw	CTTGAGAGTGTCGCTGAGC
	Rv	CAGGCGCGGATCTCTAATG
C, T, L	Fw	CCTCACCGATACCGAACGAG
	Rv	GTCAGCAGGATAGGGCTACC
C, T, R	Fw	CAAAGGCGACCACGATGATG

	Rv	CGGTCCGCGATCCTTAATCATG
C, M, R	Fw	GTGCCTTGGTGGAACGTAGTC
	Rv	GAAGCTGCGCAAGGTTTCAAG
C, O, R	Fw	GGCAAAGACAGCGCTATTCG
	Rv	CGTGTCCGATCCAGGTTAATTG
P, O, L	Fw	CTGGGATTCCGGCGTAGAAC
	Rv	CTGGCTCATGGTCACCAACATAG
P, T, L	Fw	CTCTTTGACCTCGGCTTGAG
	Rv	CGCAGCCGTTAGGACAAATG

Primer sets for *E. coli*

Name	Direction	Sequence 5' - 3'
C, O, L	Fw	ACCATAACCAGTCGCAGGTC
	Rv	CGTCAACATGATGGAAGTGG
C, M, L	Fw	GCTTCAAAACCAGCTTCGTC
	Rv	GAGCTGGCAAAACTTTCTGG
C, T, L	Fw	CGCCAGCAGTTCCTCTTTAC
	Rv	CTAACTGCGGAACCTTGAGC
C, T, R	Fw	ATGGCTTTACCGTGGATGAG
	Rv	GCTTCGCCTGTCAGGTCTAC
C, M, R	Fw	GTGTTGCTGCTTGATGTCGT
	Rv	GCAATGGCATACAGTGGTTG
C, O, R	Fw	CTCACGACAGCCTGATTGAA
	Rv	CAGGCAGGCAATCTTAAAGC
